# A rare case of primary malignant small cell carcinoma combined with urothelial cell carcinoma in the ureter

**DOI:** 10.1186/1477-7819-11-181

**Published:** 2013-08-08

**Authors:** Hoon Jang, Seung Mo Yuk, Jong Ok Kim, Dong Seok Han

**Affiliations:** 1 Department of Urology, The Catholic University of Korea Daejeon St. Mary’s Hospital, Daeheung-dong, Jung-gu, Daejeon, 301-723, Korea; 2 Department of Pathology, The Catholic University of Korea Daejeon St. Mary’s Hospital, Daeheung-dong, Jung-gu, Daejeon, 301-723, Korea

**Keywords:** Adjuvant treatment, Small cell carcinoma, Ureter, Urothelial cell carcinoma

## Abstract

**Background:**

Extrapulmonary small cell carcinomas have been reported in a variety of organs, and their incidence in the genitourinary tract is second only to that in the gastrointestinal tract. To date, however, only a few cases of small cell carcinoma of the ureter have been reported. Because the extreme rarity of this type of carcinoma, its clinical behaviour, diagnostic methods, and effective treatment modalities have not yet been determined.

**Case presentation:**

A 59-year-old man presented with a 1-month history of painless gross haematuria. Urine cytopathology revealed a urothelial carcinoma and computed tomography revealed left hydronephroureterosis with a distal ureteral stone and a mildly enhanced fungating mass just below the stone-impacted site. The preoperative TNM stage was T2N0M0. The patient underwent simultaneous diagnostic ureterorenoscopy and left laparoscopic nephroureterectomy with bladder cuff resection. Gross examination showed a 3.5 × 3.0 × 0.8 cm white, partly yellow mass in the left distal ureter. Light microscopy showed a small cell carcinoma, overlaid on a urothelial carcinoma *in situ*, invading the ureter and external lateral resection margins. The small cell carcinoma was diffusely positive for neuron-specific enolase, and exhibited focal positivity for CD 56, synaptophysin, chromogranin and cytokeratin 20. The patient was treated with adjuvant chemotherapy, consisting of cisplatin and etoposide, and radiation therapy, and has been well, without evidence of tumour recurrence or metastasis in the 10 months after surgery.

**Conclusion:**

Small cell carcinoma of the ureter is rare. Although its clinical behaviour and diagnostic modalities have not been determined and it has yet to be diagnosed immunohistopathologically, multimodality treatment including surgery, chemotherapy and radiotherapy may improve patient survival.

## Background

Although most small cell carcinomas (SCCs) originate in the pulmonary system, extrapulmonary SCCs (ESCCs) have been reported in a variety of organs [1], with genitourinary ESCCs being second in incidence only to gastrointestinal ESCCs [2]. ESCCs of the ureter are exceedingly rare, with only a few individual cases reported in the literature [3,4]. The histogenesis of this tumour remains uncertain, although several hypotheses have been suggested [5,6]. Moreover, owing to its rarity, its clinical behaviour and appropriate treatment have not been well established. We describe the clinical characteristics, pathological features and immunohistochemical findings of a primary malignant SCC combined with urothelial carcinoma.

## Case presentation

In December 2011, a 59-year-old man with an unremarkable medical history presented at the outpatient clinic with a 1-month history of painless gross haematuria. He did not complain of voiding symptoms, such as frequency, hesitancy or dysuria. He had a smoking history of one pack (Cigarettes, 20/pack) per day for 30 years, but he denied having any respiratory symptoms. No remarkable findings were noted on physical examination, and most laboratory tests yielded results within normal limits. However, the patient’s serum creatinine concentration was elevated, a urinalysis showed many red blood cells, and his urinary cytopathology indicated a urothelial carcinoma.

**Figure 1:**
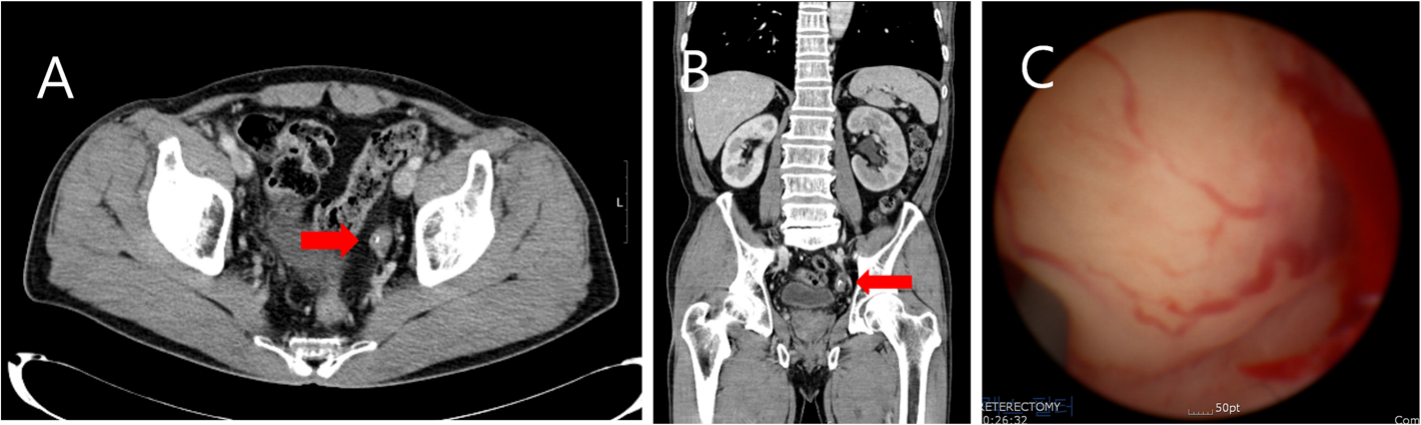
**Radiography and ureterorenoscopy. (A**,**B)**. Abdominal computed tomogram showing a stone in the left distal ureter and a mildly enhanced fungating mass lesion just below the stone-impacted site (red arrow). **(C)** Ureterorenoscopic view showing a huge, round, fungating mass with a smooth surface filling the lumen of the left distal ureter.

Chest radiography showed an old tuberculosis scar, and plain radiography of the kidneys, ureter and urinary bladder showed a radio-opaque area in the left pelvic cavity. Abdominal computed tomography (CT) revealed a right simple renal cyst, left hydronephroureterosis, a stone in the left distal ureter, and a mildly enhanced, fungating mass lesion just below the stone-impacted site (Figure 1A,B) but no evidence of pelvic lymph node enlargement. Cystoscopic examination showed a bloody urine flow in the left ureteral orifice, but no mass-like lesion.

On the basis of cystoscopic examination, imaging findings and urinary cytopathology, a urothelial cell carcinoma of the ureter, TNM stage T2N0M0, was diagnosed. We therefore performed a diagnostic ureterorenoscopy, followed immediately by laparoscopic left nephroureterectomy with bladder cuff resection. The ureterorenoscopy showed a huge, round, fungating mass with a smooth surface without papillary characteristics, filling the lumen of the left distal ureter (Figure 1C). Frozen section evaluation of a biopsy sample showed atypical urothelial cells, findings consistent with a urothelial carcinoma. Pathological examination of the tumour, removed by laparoscopic left nephroureterectomy with bladder cuff resection, showed an SCC combined with a urothelial cell carcinoma in the ureter. A bone scan and positron emission tomography showed no evidence of metastasis or remnant cancer.

After surgery, the patient was treated with four cycles of chemotherapy, consisting of 100 mg/m^2^ etoposide on days 1, 2, and 3 and 75 mg/m^2^ cisplatin on day 1, every 3 weeks. The patient also received external radiation therapy at the site of the left ureteral mass, consisting of 180 cGy per day, 5 times per week, up to a total of 30 fractions (5400 cGy) over 44 days. The patient tolerated adjuvant chemotherapy and radiation therapy well, with no significant side effects or adverse reactions, except for intermittent nausea, vomiting and generalized myalgia. He was followed up by urinalysis, urinary cytopathology, cystoscopy, an abdominal CT scan and positron emission tomography. He has been well, without evidence of tumour recurrence or metastasis, for 10 months after surgery.

### Pathology

**Figure 2:**
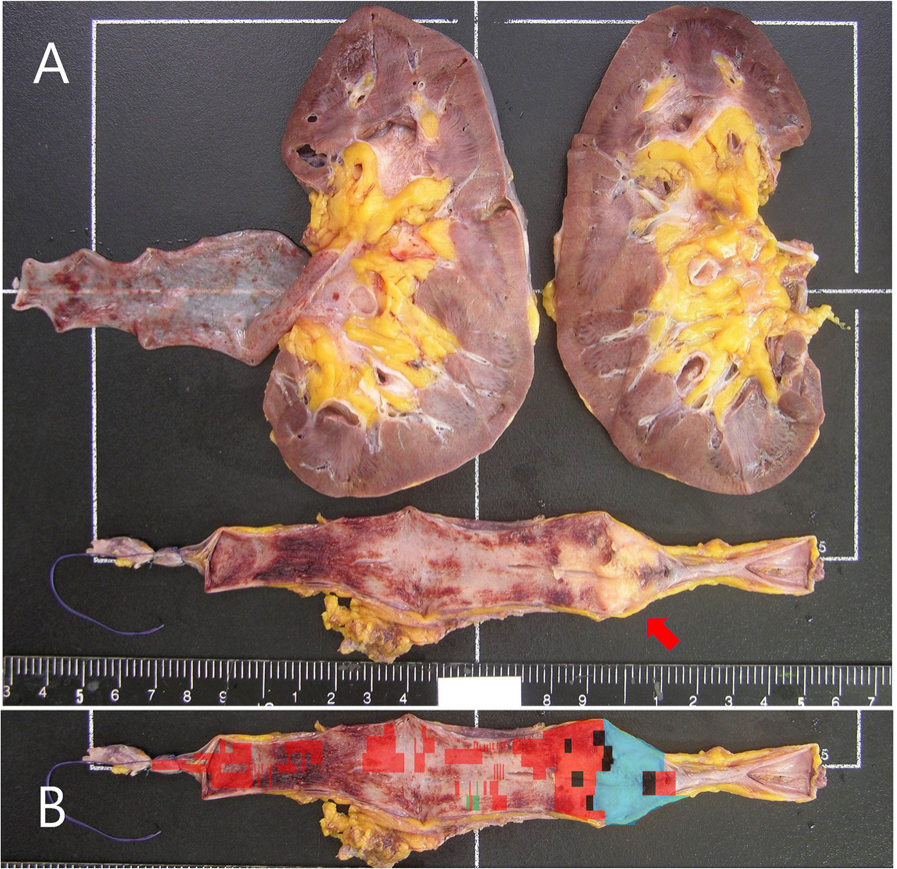
**Gross examination of the specimen and mapping of the ureter. (A)** Photograph of the separated ureter showing a poorly defined, slightly elevated mass, measuring 3.5 × 3.0 × 0.8 cm (red arrow). Its cut surface was greyish and infiltrative, and it invaded the periureteric fat beyond the muscularis propria. The mass was located 3.6 cm from the distal ureteral resection margin. The remaining mucosa was focally reddish and did not show definitive mass. The kidney contained a unilocular small cyst, 1.1 cm in diameter; the segment of the ureter attached to the kidney was dilated and did not show evidence of tumour. **(B)** Outline of the tumour, showing the distribution of the small cell carcinoma (blue), urothelial cell carcinoma *in situ* (red), invasive urothelial cell carcinoma (black) and dysplasia (green).

**Figure 3:**
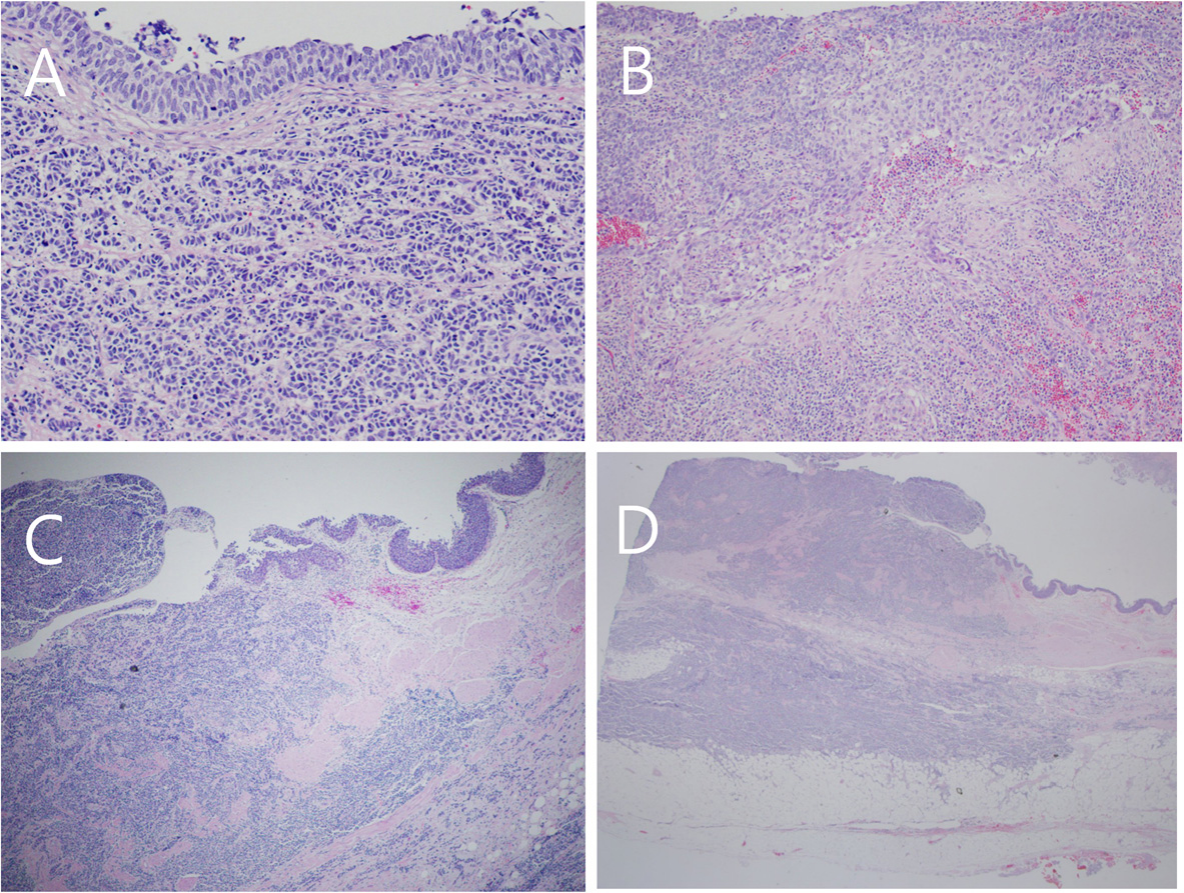
**Histochemical examination of the tumour (H & E staining). (A)** The area of small cell carcinoma showed small or round cells, with finely granular, hyperchromatic nuclei, inconspicuous nucleoli and scanty cytoplasm. Nuclear moulding and cord-like appearing cells were identified. The area of urothelial carcinoma *in situ* overlay the area of small cell carcinoma (200×). **(B)** Urothelial carcinoma cells invading the lamina propria (100×). **(C)** Juxtaposition of areas of small cell carcinoma and urothelial carcinoma, showing the small cell carcinoma invading the lamina propria as far as the periureteric adipose tissue and the overlying focal urothelial carcinoma *in situ* (40×). **(D)** Cross-sectional view of ureter showing the small cell carcinoma invading the ureter and periureteric fat and protruding into the ureteral lumen (12.5×).

Gross examination of the resected specimen showed a hydronephrotic kidney with a dilated renal pelvis and calyces, and ureteral dilation. A segment of the left ureter contained a poorly defined, slightly elevated mass (3.5 × 3.0 × 0.8 cm), which protruded into the ureteral lumen and caused near-complete obstruction of the ureter (Figure 2A). Light microscopy of the SCC area of the specimen showed small or round cells, containing finely granular, hyperchromatic nuclei, inconspicuous nucleoli and scanty cytoplasm (Figure 3A). We also observed a urothelial carcinoma *in situ* overlying the SCC, with multiple foci invading the lamina propria (Figure 3A,B). The carcinoma’s overlying mucosa was focally denuded, with the SCC exposed to the luminal surface (Figure 3C). In addition, the SCC invaded the mucosa, muscularis, periureteric fat tissue and external lateral resection margin (Figure 3D). The urothelial carcinoma *in situ* measured 15 cm in length and 3.0 cm in circumference and was located 1.4 cm from the proximal and 3.6 cm from the distal resection margin of the ureter (Figure 2B). However, there was no evidence of invasion of the perineural, lymphatic, renal parenchymal area, or of the renal artery or renal vein.

**Figure 4:**
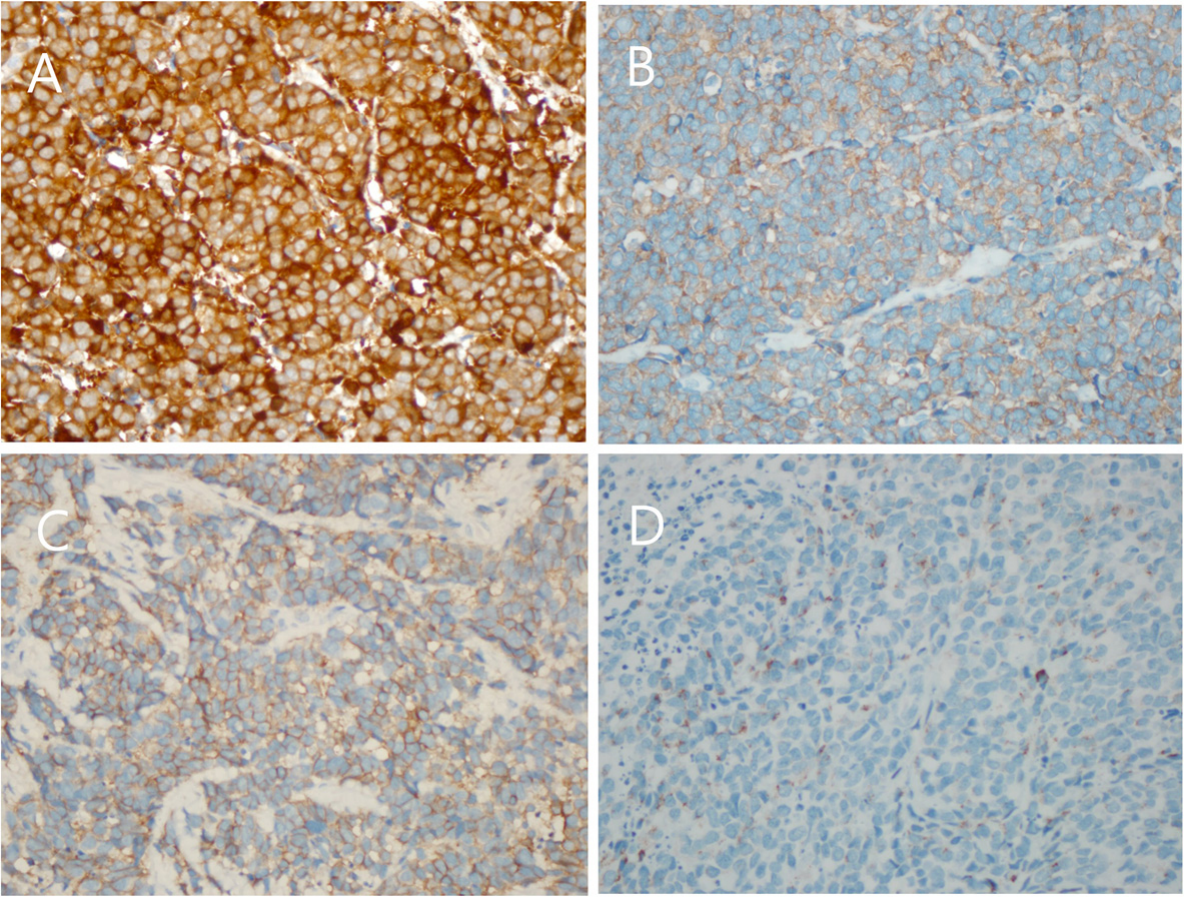
**Immunohistochemical examination of the tumour.** Immunohistochemical staining showing that the area of small cell carcinoma was positive for **(A)** neuron-specific enolase and **(B)** synaptophysin, **(C)** weakly positive for CD56, and **(D)** focally positive for chromogranin (each 400×).

Immunohistochemical staining showed that the SCC was diffusely positive for neuron-specific enolase and focally positive for CD 56, synaptophysin and chromogranin (Figure 4), whereas the urothelial cell carcinoma and urothelial cell carcinoma *in situ* were negative for those markers.

## Discussion

Neuroendocrine tumours can be broadly classified as well differentiated (true carcinoids), moderately differentiated (atypical carcinoids) and poorly differentiated (SCCs), with the latter including ESCCs and small cell lung cancer (SCLC). ESCCs account for approximately 0.1% to 0.4% of all SCCs, with the most common sites being gastrointestinal and genitourinary tracts.

Genitourinary SCCs have been reported at many sites, including the renal pelvis [7], urinary bladder [8], ureter [3,4], urethra [9] and prostate [10], with the most common sites being the urinary bladder and prostate. In general, however, these tumours are rare, with SCC of the ureter being extremely rare.

SCCs of the respiratory tract are thought to originate from neuroendocrine cells in the bronchus called amine precursor uptake and decarboxylation or Feyrter cells [11] and therefore express a variety of neuroendocrine markers. SCCs of the respiratory tract can occur in combination with other histological variants of lung cancer, including squamous cell carcinoma and adenocarcinoma, with the malignant tumour diagnosed and classified as a combined SCLC (c-SCLC) [12].

The histogenesis of SCCs of the ureter remains unclear. SCCs of the genitourinary system might originate from multipotential epithelial cells in the genitourinary tract [5] or from intrinsic neuroendocrine cells in the normal genitourinary tract derived from the neural crest during embryogenesis [6].

Our patient presented with an SCC of the ureter combined with high-grade urothelial carcinoma, in agreement with the hypothesis that these tumours originate from multipotential stem cells of the ureter. However, this SCC of the ureter was positive for several neuroendocrine markers, in agreement with the hypothesis that SCC originates from intrinsic neuroendocrine cells within the normal genitourinary tract derived from the neural crest during the embryogenesis. Further evaluation is required to clarify the pathogenesis and origin of ureteral SCCs.

Our patient visited the hospital with painless gross haematuria. The CT showed an ill-defined ureteral mass with a ureteral stone. The most common symptom of SCC of the ureter is asymptomatic gross haematuria, although flank pain with or without gross haematuria has also been reported [13]. Most patients are diagnosed with SCC of the ureter after immunohistopathological examination of samples removed at biopsy or surgery. SCCs can be diagnosed by determining the ultrastructure of secretory neuroendocrine granules and immunohistochemical positivity for synaptophysin and neuron-specific enolase. Prior to immunohistopathological examination, no clinical symptoms or imaging results can distinguish SCC of the ureter from other conditions, such as urothelial carcinoma, which causes asymptomatic gross haematuria or a ureteral stone, or inflammatory disease that causes ureteral obstruction. Urinary cytopathology and CT scanning are helpful in diagnosing and preoperative planning of treatment for asymptomatic gross haematuria. Urinary cytopathology can reveal evidence of the origin of the mass, whereas CT scanning can determine its location, size and the presence of lymph node metastasis or ureteral stones.

Our patient had a smoking history of one pack per day for 30 years. The most frequent cause of lung cancer, especially SCLC, is smoking [14,15]. Smoking has been reported to result in greater histological and genetic damage to normal epithelium in patients with SCLC than in patients with non-small cell lung cancer; allele loss is much greater in the former than in the latter [16]. As with SCLC, smoking might be the primary risk factor for SCC of the genitourinary tract; patients with SCC at any site in the urinary tract have been reported to have a history of heavy smoking. Although SCCs of the genitourinary tract may also occur in nonsmokers [5,10,17], we believe that smoking is the main risk factor for the development of ESCCs. Further clarification of the mechanism and relationship between smoking and ESCC is required.

Our patient underwent laparoscopic nephroureterectomy with bladder cuff resection, followed by adjuvant chemotherapy with cisplatin and etoposide, and radiation therapy. Because of the rarity of these tumours, standard treatment has not yet been established [18]. Multimodality therapy, including surgery, radiation and chemotherapy, has been administered previously and has been shown to be feasible with regard to improvement [7,8,18]. This multimodal therapy is based on the histological similarity of SCC of the ureter with SCLC. Effective therapeutic modalities and treatment protocols for SCC of the ureter need to be further evaluated.

The prognosis of patients with SCC of the genitourinary tract is poor, with most dying of disease within 1 year [9]. SCCs of the genitourinary tract tend to progress rapidly, invading the regional lymph nodes, liver and other organs [5]. The extent of disease at diagnosis is regarded as the most sensitive predictor of survival [2]. Fortunately, our patient remains alive, with no evidence of tumour recurrence, lymph node metastasis or distant metastasis, 10 months after surgery.

## Conclusion

We report a rare case of primary malignant SCC combined with urothelial carcinoma in the ureter. Although the clinical behaviour and methods of diagnosis of SCC of the ureter have not been established, preventing diagnosis prior to immunohistopathological examination of biopsied or resected samples, multimodal therapy including surgery, chemotherapy and radiotherapy may improve patient survival. Urologists should be aware of the possibility of SCC in the ureter in patients presenting with a ureteral mass. Further prospective studies and standardized diagnostic criteria are needed to clarify the epidemiology, diagnostic tools and most effective treatment protocol for SCC of the ureter.

## Consent

Written informed consent was obtained from the patient for publication of this case report and any accompanying images. A copy of the written consent is available for review by the editor-in-chief of this journal.

## Abbreviations

CT: Computed tomography; ESCC: Extrapulmonary small cell carcinomas; H & E: Haematoxylin and eosin; SCC: Small cell carcinoma; SCLC: Small cell lung cancer.

## Competing interests

The authors declare that they have no competing interests and that there is no financial competing interest.

## Authors’ contributions

HJ and SMY carried out the laparoscopic nephroureterectomy and drafted the manuscript. JOK performed the immunohistopathological experiments. DSH formulated the adjuvant radiochemotherapy protocol and supervised the study. All authors read and approved the final manuscript.
